# The HAPPY study (Holistic Approach to Pregnancy and the first Postpartum Year): design of a large prospective cohort study

**DOI:** 10.1186/1471-2393-14-312

**Published:** 2014-09-08

**Authors:** Sophie EM Truijens, Margreet Meems, Simone MI Kuppens, Maarten AC Broeren, Karin CAM Nabbe, Hennie A Wijnen, S Guid Oei, Maarten JM van Son, Victor JM Pop

**Affiliations:** Department of Obstetrics and Gynaecology, Máxima Medical Centre, Veldhoven, De Run 4600 The Netherlands; Department of Medical and Clinical Psychology, Tilburg University, Warandelaan 2, Tilburg, P.O. BOX 90153, Tilburg, 5000 LE The Netherlands; Department of Obstetrics and Gynaecology, Catharina Hospital, Michelangelolaan 2, Eindhoven, The Netherlands; Laboratory of Clinical Chemistry and Haematology, Máxima Medical Centre, Veldhoven, De Run, 4600 The Netherlands; Clinical Laboratory, Diagnostiek voor U, Stratumsedijk 28a, Eindhoven, The Netherlands; Department of Midwifery Science, Maastricht-ZUYD, Universiteitssingel 60, Maastricht, The Netherlands; Department of Clinical and Health Psychology, Utrecht University, Padualaan, 14 Utrecht The Netherlands

**Keywords:** Pregnancy, Maternal well-being, Trimester specific symptoms, Distress, Depression, Thyroid function, Delivery, Childbirth, Postpartum, Breastfeeding

## Abstract

**Background:**

The HAPPY study is a large prospective longitudinal cohort study in which pregnant women (N ≈ 2,500) are followed during the entire pregnancy and the whole first year postpartum. The study collects a substantial amount of psychological and physiological data investigating all kinds of determinants that might interfere with general well-being during pregnancy and postpartum, with special attention to the effect of maternal mood, pregnancy-related somatic symptoms (including nausea and vomiting (NVP) and carpal tunnel syndrome (CTS) symptoms), thyroid function, and human chorionic gonadotropin (HCG) on pregnancy outcome of mother and foetus.

**Methods/design:**

During pregnancy, participants receive questionnaires at 12, 22 and 32 weeks of gestation. Apart from a previous obstetric history, demographic features, distress symptoms, and pregnancy-related somatic symptoms are assessed. Furthermore, obstetrical data of the obstetric record form and ultrasound data are collected during pregnancy. At 12 and 30 weeks, thyroid function is assessed by blood analysis of thyroid stimulating hormone (TSH), free thyroxine (FT4) and thyroid peroxidase antibodies (TPO-Ab), as well as HCG. Also, depression is assessed with special focus on the two key symptoms: depressed mood and anhedonia. After childbirth, cord blood, neonatal heel screening results and all obstetrical data with regard to start of labour, mode of delivery and complications are collected. Moreover, mothers receive questionnaires at one week, six weeks, four, eight, and twelve months postpartum, to investigate recovery after pregnancy and delivery, including postpartum mood changes, emotional distress, feeding and development of the newborn.

**Discussion:**

The key strength of this large prospective cohort study is the holistic (multifactorial) approach on perinatal well-being combined with a longitudinal design with measurements during all trimesters of pregnancy and the whole first year postpartum, taking into account two physiological possible markers of complaints and symptoms throughout gestation: thyroid function and HCG. The HAPPY study is among the first to investigate within one design physiological and psychological aspects of NVP and CTS symptoms during pregnancy. Finally, the concept of anhedonia and depressed mood as two distinct aspects of depression and its possible relation on obstetric outcome, breastfeeding, and postpartum well-being will be studied.

## Background

### Antenatal physical and mental well-being

Pregnancy is a period during which a woman faces a substantial number of physiological changes of the body, commonly together with psychological changes. These changes often result in complaints and symptoms which are typical to pregnancy. There are two important pregnancy-related complaints or symptoms that are frequently observed and regarded as consequences of substantial hormonal changes during gestation: nausea, vomiting and symptoms of carpal tunnel syndrome.

*Nausea and vomiting during pregnancy (NVP)* is a common phenomenon (50-80% of pregnancies with variable severity [1]) and has been related to both psychological and physiological factors, including high levels of the hormone human chorionic gonadotropin (HCG) and thyroid hormone levels. In its extreme form, NVP or ‘morning sickness’ can manifest itself as hyperemesis gravidarum (HG), a potentially life threatening but rare condition (0.5-2%) which is characterised by nausea, vomiting, severe dehydration and weight loss resulting in hospitalisation [2]. Although most cases of HG are related to extremely high titres of HCG (>200,000 IU/l), there have been some reports that high levels of maternal thyroid hormone (FT4) or low levels of thyrotropin (TSH) often reported as thyrotoxicosis might also predispose to more severe symptoms of NVP [3]. However, studies that investigated in one design both HCG and thyroid function, as well as psychological background features of NVP, have not been reported.

*Carpal tunnel syndrome (CTS)* is common during gestation and occurs because of compression of the median nerve in the wrist in one or both hands, leading to a tingling sensation, numbness and sometimes pain. Especially hormonal changes [4], and fluid retention [4, 5] have been suggested as contributing factors to development of CTS in pregnancy. CTS occurs most frequently during the third trimester of pregnancy and a majority of women have symptoms that are severe enough to affect hand function and sleep [6], resulting in impaired quality of life [7, 8]. Accurate data of prevalence rates are difficult to find in the literature and vary substantially (1-60%) [9–11]. This is mainly explained by the fact that most women do not report the symptoms, physicians do not ask for them and – possibly due to the conservative attitude towards treatment during pregnancy – most women with (substantial) CTS symptoms do not receive electrodiagnostic examination (EDE) [12]. However, the Boston Carpal Tunnel Questionnaire (BCTQ) [13] has shown to be a highly reliable and sensitive self-rating questionnaire for CTS [14, 15]. Moreover, it is a user-friendly instrument which can easily be used during pregnancy to assess the occurrence of CTS. An underactive thyroid, called hypothyroidism (HT), has been long time reported to be associated with increased risk of CTS. In a systematic review [16], the pooled odds ratio of HT patients of CTS was 1.4 indicating a 40% higher prevalence rate of CTS in patients with HT compared to healthy controls. HT might produce alterations of fluid balance and peripheral tissue oedema resulting in nerve compression in the carpal tunnel. A possible relation between CTS symptoms and thyroid function during gestation has not been reported in the literature yet. Apart from these physiological changes, a considerable proportion of pregnant women experiences psychological distress, varying from pregnancy-related worries to general symptoms of anxiety and depression. The occurrence of distress during pregnancy is subject to much debate: is there an increasing number of women suffering from depression and anxiety throughout gestation? The literature is inconclusive, mostly because there are very few studies that repeatedly assessed anxiety and depression. Most studies measured these symptoms only once during pregnancy, a few studies twice, but the systematic review of Bennett et al. [17] and the review of Alder et al. [18] found no appropriate studies that assessed symptoms of depression and anxiety during all trimesters of pregnancy. Furthermore, some researchers suggested symptoms of depression and anxiety to be stable throughout pregnancy [19], while others stated that maternal mood and well-being fluctuate across pregnancy [20]. With regard to depression, there is growing awareness that the two key symptoms of depression (depressed mood and anhedonia) are two distinct entities with possibly different impact on health [21, 22]. Depressed mood is more and more regarded as a state, while anhedonia is regarded as a trait factor of depression. Although this distinction has become increasingly apparent in psycho-cardiology [23, 24] (it is especially anhedonia that has been related to poor cardiovascular outcome rather than depressed mood [24]), this concept has hardly been investigated in perinatal research. As a consequence, conflicting data have been reported on a possible relation between antenatal mood problems and obstetric outcome. The review of Dunkel Schetter and Tanner (2012) concluded that anxiety, depression and stress during pregnancy are related to obstetric complications and adverse pregnancy outcomes, like preterm birth and low birth weight [25]. However, a meta-analysis in 2007 with 50 studies found no significant evidence of an association between symptoms of anxiety during pregnancy and adverse perinatal outcomes [26], and a more recent meta-analysis with 35 studies found a significant but negligible to small relationship between stress during pregnancy and negative birth outcomes [27]. These conflicting findings are mainly to be explained by poor methodology: regarding definition and assessment of distress, no repeated assessments during pregnancy, and lack of adequate sample power. Further research on depression, anxiety, and stress in the different stages of pregnancy (with special attention to the concept of anhedonia) is therefore required, to investigate a possible relationship with adverse birth outcomes.

Literature also shows distress during pregnancy to be related to increased request of epidural analgesia during labour [28, 29]. In The Netherlands, only a minority of women asks for epidural analgesia during delivery, although this number is increasing: from 6% in 2004 to 18% in 2012 [30, 31]. On the other hand, epidural analgesia has been associated with increasing numbers of instrumental delivery (hormonal stimulation because of weakening of contractions, or ventouse) [32]. Studies, evaluating whether the request of epidural analgesia is related to pregnancy-related distress and whether the changes in distress over time influence the request of epidural analgesia, have hardly been published.

Concerning the long-term effects, there is growing evidence that pregnancy distress might interfere with infant development and there are studies showing long-term effects of maternal distress throughout the childhood and adolescence [33, 34].

### Maternal and foetal thyroid function during pregnancy

Adequate maternal thyroid function is extremely important during pregnancy, not only for the developing foetus but also for optimal obstetric outcome. Up to 10% of the women of fertile age have antibodies against thyroid peroxidase enzyme (TPO-Ab). The TPO-Ab are involved in the autoimmune thyroiditis process resulting in an inactive thyroid. These women have a 4 fold increased risk for abortion and a 15–20 fold increased risk to develop postpartum thyroid dysfunction [35, 36]. Impaired thyroid function – especially high TSH referring to subclinical hypothyroidism – has been related to preterm birth, intrauterine growth restriction (IUGR), small for gestational age (SGA), and pre-eclampsia [37]. Maternal thyroid hormone is especially important for the developing central nervous system (CNS) of the foetus. However, as shown in a recent review, most studies looking at foetal outcome and maternal thyroid function did not use intra-uterine foetal parameters to evaluate the CNS development, such as ultrasound data [38]. In The Netherlands, as part of antenatal screening, every pregnant woman receives a standardized and extremely well protocolized ultrasound (SEO) at 20 weeks of pregnancy. During the SEO, the foetal CNS is a special focus of interest including assessments of ventricle size, cerebellum size, and spine development. Also the femur length, abdominal circumference, head circumference, and biparietal diameter are measured as objective parameters of foetal growth. There are very few data on the relation between maternal thyroid function and SEO data. Moreover, maternal thyroid dysfunction has been related to abnormal foetal position such as breech and problematic cephalic position, both in turn related to obstetrical complications [39, 40]. Also, thyroid dysfunction in general – during pregnancy and the postpartum period – has been related to mood disorders [41, 42]. The mechanisms behind these associations are unclear. This is partly to be explained because some associations have been found in large epidemiological studies that were not intended to investigate a specific role of thyroid dysfunction on obstetric outcome and as a consequence a substantial number of confounders were not taken into account [43]. Other studies with an adequate design suffered from poor epidemiological power (few cases of thyroid dysfunction and obstetric complications) [44]. Large prospective studies in which thyroid function and psycho-social characteristics have repeatedly been assessed during pregnancy are scarce. Moreover, adding a biological variable (thyroid function) to a model with predominantly psychological variables (maternal depression and anxiety during pregnancy), may elucidate on the multifactorial origin of impaired infant development as has been published before by our research group [45, 46].

### The early postpartum period

#### Neonatal thyroid function

After birth, there is a neonatal TSH surge to stimulate neonatal thyroid function in order to start brown fat production for thermogenesis. The umbilical blood vessels are thought to accurately reflect neonatal thyroid function before the TSH surge starts and assessment of thyroid function in the umbilical cord will reflect maternal thyroid hormone function during the last trimester [47, 48]. As in most Western countries, in The Netherlands, there is a nationwide congenital screening program of neonatal thyroid function between three to five days postpartum. Until now, little is known about the relation between maternal thyroid hormone levels in pregnancy, umbilical cord levels, and neonatal heel screening results. Within the HAPPY study, pairs of maternal and neonatal thyroid data will be sampled to investigate whether poor maternal thyroid function has neonatal thyroid function consequences. From previous research in the same area it is known that because of logistic difficulties (home delivery, referral during labour from home to hospital, delivery in different hospitals) in about 70% of the participating women umbilical cord blood will be obtained [47].

### Maternal well-being and infant development during the postpartum period

Up until now, it is unknown how long the period lasts that a woman normally needs to recover from pregnancy and delivery. Similar to pregnancy, there is hardly any insight into complaints and symptoms that occur during the postpartum recovery period: what are typical complaints and symptoms for this period, how long does it last, when does it end, is it related to feeding sessions at night, or to complicated delivery? As a consequence, many physiological signs and symptoms which seem to be typical for the postpartum period (cognitive and sleeping problems) are incorrectly related to postpartum depression, which is generally regarded as a stigmatizing condition. Postpartum mood disorders mostly refer to postpartum blues (occurring in 40% to 80% of the women preferentially during the first postpartum week) and postpartum depression with prevalence between 13-19% [49]. The negative effect of (especially ‘chronic’) maternal depression on the developing child has extensively been described [50–53]. Depressed mothers may become apathetic, emotionally withdrawn, and unresponsive to their offspring [52], while the first months of the newborn’s life are the ‘sensitive period’ for the mother-child bonding [53]. Maternal postpartum depression is harmful to the development of the newborn, whose early socialization, cognitive development, and speech acquisition are largely in mother’s hands [52].

However, research that assesses maternal distress on a regular basis during pregnancy and during a substantial period of time during the postpartum period is scarce. Furthermore, it is interesting to investigate possible differences between the impact of distress at different time points during gestation (first versus third trimester) on infant development.

#### Breastfeeding

The WHO and UNICEF have rather strict criteria defining optimal patterns of breastfeeding: *1) early initiation of breastfeeding within one hour of birth; 2) exclusive breastfeeding for the first six months of life; and 3) the introduction of nutritionally-adequate and safe complementary (solid) foods at six months together with continued breastfeeding up to two years of age or beyond* [54] *.* However, many infants and children do not receive optimal feeding. For example, only about 38% of infants aged up to six months worldwide are exclusively breastfed [54]. In The Netherlands, about 75% of the women start breastfeeding after childbirth but almost half of them has stopped within the first three months postpartum and less than 25% still breastfeed at six months postpartum [55, 56]. In Norway and Sweden, more than 95% of the women start breastfeeding and 67-80% of these Scandinavian women still breastfeed at six months postpartum [57, 58]. Determinants that predict start and continuation of breastfeeding are of multifactorial origin including educational level, socioeconomic status, marital status, partner involvement, and maternal distress during gestation and postpartum [59]. Also, facilities at work to breastfeed [60] and the perceived milk supply [61] influence the duration of breastfeeding. When women are depressed during pregnancy, they are less likely to initiate or to maintain breastfeeding [62, 63]. However, the relation between postpartum depression and breastfeeding remains equivocal [63]. There is still the dispute whether women who discontinue breastfeeding are at increased risk for postpartum depression or vice versa: are women with postpartum depression at risk for poor breastfeeding ‘behaviour’? It is important to detect women who experience difficulties with the challenges of breastfeeding (e.g. low milk production, infant weight loss while being exclusively breastfed, etc.) despite their willingness and continuous efforts to try to breastfeed their infants. Antenatal intervention techniques (including involvement of the partner in decision making) can increase the likelihood to breastfeed [64].

Moreover, the effect of epidural analgesia during labour on the start and continuation of breastfeeding during the early postpartum week is poorly understood. A recent study showed a 26% increased risk of not initiating breastfeeding after epidural anaesthesia in primiparous women who delivered vaginally [65]. Similarly, it has long been noticed that oxytocin also has an impact on the CNS, and oxytocin and vasopressin are hypothesized to integrate social information into attachment processes. There is growing evidence that oxytocin might be useful as an antipsychotic drug in the future. These days, synthetic oxytocin is the most common medical intervention in childbirth, despite the great variety of plausible side effects [66]. There are theoretical reasons to test whether the increasing incidence of breastfeeding difficulties and the earlier than desired cessation of breastfeeding are related to the use of synthetic oxytocin during labour [66]. A recent study from Spain showed an odds ratio for bottle-feeding of 1.45 (95% CI 1.29-1.64) and of 2.29 (95% CI 1.41-3.74) for withdrawal of breastfeeding at three months in those women who received oxytocin during labour [66]. However, because oxytocin is often given in case of weakness of contractions which occurs frequently in women who ask for epidural anaesthesia, this request being partly to be explained by psychological characteristics, it is obvious that investigating a possible relation between oxytocin use and breastfeeding patterns needs a multifactorial design.

### Study objectives and hypotheses

The HAPPY study (Holistic Approach to Pregnancy and the first Postpartum Year) contains several aims with regard to both pregnancy and the postpartum period.

**Figure 1:**
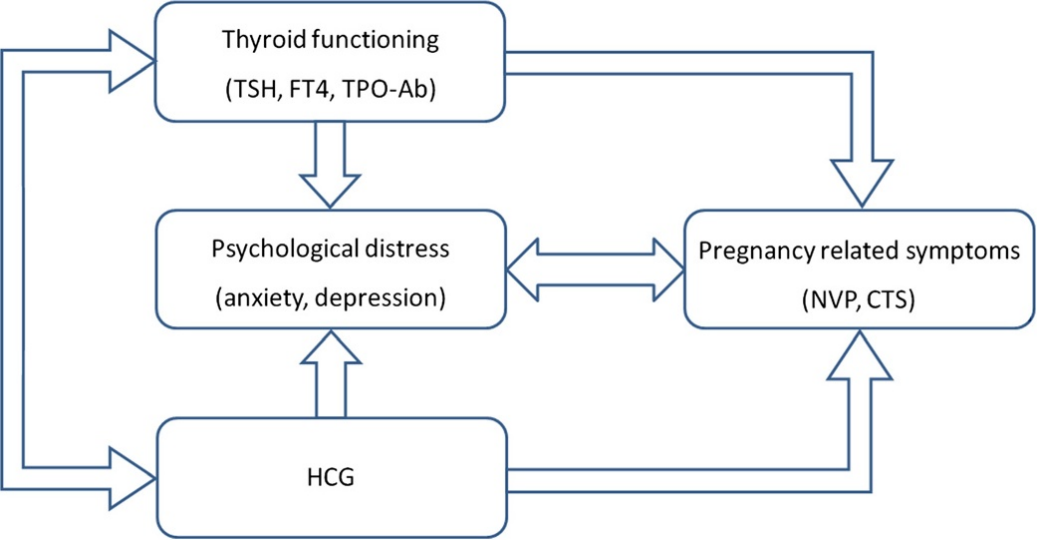
**Multifactorial model with hypothesized associations between physiological and psychological factors during pregnancy.**

The primary aim of HAPPY concerning *pregnancy* is to measure the prevalence and changes in pregnancy-related complaints and symptoms over time, with special focus on NVP and CTS. Both a psychological and a physiological model will be tested to explain the variance of (severe) NVP and CTS symptoms (Figure 1).

Also, the occurrence of maternal distress (anxiety and depression) is assessed during different trimesters. Secondly, the changes in thyroid function are measured over time during pregnancy and we will investigate a possible independent effect of thyroid dysfunction on foetal development as assessed by a standardized ultrasound protocol at 18–22 weeks gestation. Moreover, a possible independent effect of thyroid dysfunction on maternal mood will be investigated as well as on obstetric outcome including abnormal foetal position at term, the prevalence of preterm birth (<37 weeks of gestation) and its possible causes such as preterm premature rupture of membranes (PPROM), the occurrence of pre-eclampsia and other obstetric complications.

Apart from collecting observational data, the following hypotheses will be tested:

- Both physiological (HCG and thyroid hormone) and psychological parameters predict the occurrence of (severe) NVP symptoms
- Women with suboptimal thyroid function (reflected by high TSH levels) are at particular risk of developing (severe) CTS symptoms during the last trimester
- Women with high TSH and/or high titres of TPO-Ab during early gestation are at particular risk for poor foetal neurologic development assessed by ultrasound at 20 weeks gestation
- Women with subclinical hypothyroidism are at risk for developing pre-eclampsia, IUGR, SGA and preterm birth
- Chronic depression during gestation is predominantly to be explained by anhedonic symptoms at baseline (first trimester) rather than depressed mood
- It is the anhedonic mood rather the depressed mood of the concept of depression that interferes with poor obstetric outcome
- Distressed women in particular ask for epidural analgesia at delivery.

With regard to the *postpartum period*, the primary aim is to investigate the effect of chronic depression during pregnancy on postpartum recovery. Secondary outcome is the impact of thyroid autoimmune disease on postpartum depression. Tertiary outcome is the relation between psychological determinants and initiating and continuation of breastfeeding. The following hypotheses have been developed regarding the postpartum period:

- Women with an auto-immune compromised thyroid function (as reflected by the presence of elevated titres of TPO-Ab assessed during pregnancy) are at risk for developing postpartum depression
- Within the concept of depression, it is anhedonia rather than depressed mood assessed during pregnancy that predicts the occurrence of (chronic) postpartum depression
- Women with high levels of distress during pregnancy and postpartum are at risk for poor breastfeeding figures (both starting and continuation)
- Women who receive epidural analgesia during labour are at risk for poor breastfeeding figures during the first postpartum week.

## Methods/design

### Design and setting

The HAPPY study is a population-based prospective longitudinal cohort study from early pregnancy up to the end of the first post-partum year. The HAPPY study is conducted in South-East Brabant (southern part of The Netherlands), in the Eindhoven area. South-East Brabant is defined as semi-rural and about 7,000 babies were born in this area in 2012. Seventeen community midwife practices (consisting of 55 midwives) consented to participate in the recruitment of participants. Furthermore, the two obstetric departments of two large hospitals in the area collaborate. The care providers of the obstetric departments collect umbilical cord blood after delivery and provide the fully completed obstetric record forms for data analysis after written informed consent of the participants. The Primary Care laboratory of Eindhoven (Diagnostiek voor U) is responsible for collection of the blood samples and the ultrasounds. The Laboratory of clinical chemistry and haematology of the Máxima Medical Centre Veldhoven is responsible for analysis of TSH, FT4, TPO-Ab and HCG and storage of the blood samples.

### Recruitment

Dutch-speaking Caucasian women (or third generation women of other ethnic groups) who have their first prenatal visit between January 2013 and September 2014 are eligible to participate in the study from the early beginning of their pregnancy. Exclusion criteria in this study are: gemelli pregnancy (or higher order pregnancies), endocrine disorder, use of thyroid medication, severe psychiatric disease (schizophrenia, borderline, or bipolar disorder), HIV, drug or alcohol addiction problems, or any other disease resulting in treatment with drugs that are potentially adverse for the foetus and need careful follow-up during pregnancy. Eligible pregnant women (and their partner) receive written and oral information about the study at their first prenatal visit. Recruitment takes place by the midwives of 17 primary care community midwife practices in the area of South-East Brabant, from January 2013 until September 2014.

The Dutch obstetric care system is organised in primary care - represented by independent midwives providing care to women with low-risk pregnancies - and secondary care, represented by hospital midwives and gynaecologists who are responsible for high-risk pregnancies. Management of 84% of all pregnant women starts in midwifery practices [31]. The other 16% are high-risk. The high-risk population consists of women with gemelli pregnancy or with chronic diseases such as diabetes, thyroid dysfunction, hypertension, or psychiatric disorders; all exclusion criteria for participation. As 84% [31] of all pregnant women will have at least one prenatal visit in a primary care midwifery practice (usually a first antenatal control between six to ten weeks of gestation), recruitment takes place by the primary care community midwives. If women decide to participate, written informed consent is obtained. During regular blood assessments (10–12 weeks and 26–30 weeks of gestation), an additional tube of blood is withdrawn for thyroid function and HCG analysis. This means that no additional interventions are needed. Written informed consent includes participation in the study and inclusion of all obstetric data from the medical record form of the community midwife and obstetrician (including ultrasound data), a detailed delivery description and neonatal condition, and data of newborn screening (heel prick test).

The HAPPY study is approved by the Ethical Board of Tilburg University (with special attention to the type of questionnaires that are used) and has been evaluated by the Medical Ethical Committee of the Máxima Medical Centre Veldhoven. Because all additional blood samples were obtained during regular blood assessments as part of regular obstetric care, the board confirmed that no additional approval of a medical committee was needed.

### Sample size calculation

The total amount of pregnancies in the area of the 17 community midwife practices is about 5,000 per year. With 10-15% non-Caucasian, 10-15% early abortion rate and 1.7% gemelli pregnancies, approximately 3,700 pregnant women will be eligible annually. Based on previous pregnancy-related research experiences of the last 20 years in this area, the response rate is expected to be high (70%) especially when no additional blood tests or interventions have to be performed. This will result in about 2,600 inclusions annually. During pregnancy, the rate of women moving outside the area is in general low which means that a number of 2,500 women are expected to finish the study protocol until delivery. After a pilot period of three months followed by a running-in period of three months, it is estimated that all midwife practices will collaborate within six months. Therefore, the period of recruitment will take about 18 months to include 2,600 women.

Because different types of events with different prevalence rates might interfere with the sample size needed for appropriate statistics, the number of 2,500 women is estimated to meet the standard epidemiological criteria. With 2,500 participants, the following numbers of abnormal events are estimated to be found: 108 women with a foetus in breech position (4.3%) [31], 185 preterm births (7.4%) [31], 450 women (18%) [67] will develop depression during pregnancy and 325–475 women (13-19%) will develop postpartum depression [49, 67] during the first postpartum year. According to thyroid function, we will find 200 (8%) women with elevated titres of TPO-Ab and 100 with TSH > 2.5 IU/l [36]. These numbers will allow statistical analysis with sufficient power. According to Pocock [68], with an expected prevalence rate of 4-10% (breech, preterm birth, TPO antibodies, TSH > 2.5), a significance level of 5% and a power of 80%, 1,500-2,140 women need to be included (depending on the expected prevalence rate). In order to anticipate on (low) drop out, 2,600 women will be included.

### Data collection

**Figure 2:**
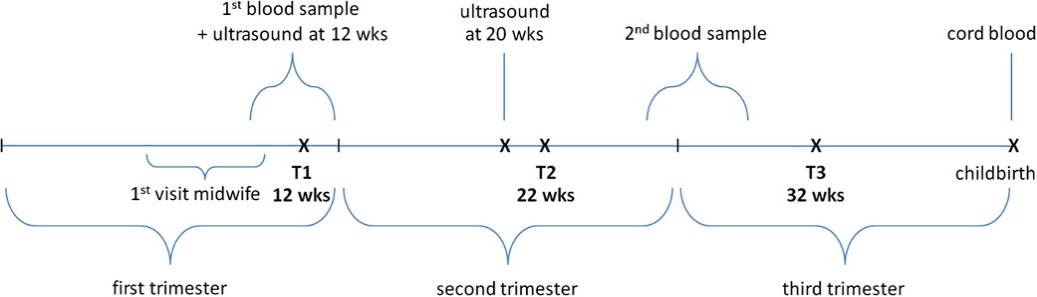
**Timetable of assessments during pregnancy.**

Questionnaire assessments *during pregnancy* are planned in each trimesters of pregnancy (T1-T3, Figure 2). Participants receive the questionnaires by postal mail or internet according to the woman’s personal choice. If a woman does not respond within one week, a researcher of the study team (CB), will call her to inform whether she received the questionnaire and check her email or postal address if this was not the case. After checking the contact information, the women will receive a reminder email (or reminder call). If a woman does not respond – despite having received a written informed consent – she is regarded as lost to follow-up. If obstetric data that are collected by questionnaires during pregnancy are missing (parity, previous abortion e.g.) these data will be collected from the parturition record form which contains all relevant obstetric data.

Apart from a previous obstetric history, the questionnaires include demographic features, assessment of distress symptoms and somatic symptoms which are frequently mentioned during physiological gestation. During pregnancy, the obstetrical data from the obstetric record form and ultrasound data (at 12 and 20 weeks of gestation) are collected.

Furthermore, at standardized blood assessment around 10–12 weeks and 26–30 weeks of gestation an additional tube of blood is withdrawn for thyroid function assessment and HCG analysis.

If collection of an additional tube of blood fails, these data (thyroid parameters and HCG) will be regarded as missing, because the study protocol does not allow to perform an additional venipuncture.

After childbirth, all obstetrical data with regard to start of labour, mode of delivery and complications are collected. Moreover, cord blood is collected after cutting of the umbilical cord, to be used for neonatal thyroid function assessment, which accurately represents foetal thyroid function. Since the additional tube of blood is drawn during the regular blood assessment (which already needs a venipuncture), and the umbilical cord and the placenta normally are thrown away for destruction after birth, it can be stated that there are no additional interventions performed to either mother or the newborn within the HAPPY study.

**Figure 3:**
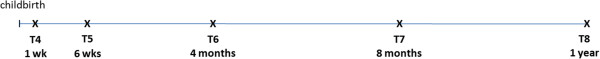
**Timetable of assessments during the first postpartum year.**

In the *postpartum period*, questionnaire assessments have been planned at one and six weeks and at 4, 8, and 12 months postpartum (T4-T8, Figure 3).

### Materials/measures

#### Obstetric history, perinatal measures and obstetrical outcomes

The obstetric record form which is standardized in our country is used to collect all relevant data of pregnancy and delivery from the community midwife and/or the obstetrician. Obstetric data like blood pressures during pregnancy, gestational age at birth, usage of anaesthesia, mode of delivery, duration of labour, complications, and birth weight are carefully assessed by the care providers.

#### The standardized 20 weeks ultrasound (SEO)

The following data – assessed during the SEO – are used in HAPPY: the biparietal diameter, ventricle size, cerebellum size, and femur length. Data of ultrasounds performed at other time points during gestation are also collected.

#### Blood analysis of thyroid function and HCG

TSH, FT4, TPO-Ab, and HCG are measured in Li-heparin plasma using electrochemoluminescence assays (Cobas® e 601, Roche Diagnostics, Mannheim Germany). The non-pregnant reference range of TSH is 0.4-4.0 mU/L, of FT4 10.0-24.0 pmol/L, and of TPO-Ab < 35 kU/L. The reference ranges of TSH and FT4 at the first and third trimester will be defined in TPO-Ab negative women using the 2.5th and 97.5th percentiles as lower and upper limit of normal thyroid function.

#### Questionnaires

At eight time points during the period between 12 weeks gestation and 12 months postpartum, the participants receive several questionnaires as shown in Table 1.

**Table 1:** **Time points of assessments during pregnancy and the first postpartum year**

Concept	Instrument	Time points during pregnancy (T1-T3)			Time points in the first postpartum year (T4-T8)				
		T1	T2	T3	T4	T5	T6	T7	T8
		12wk	22wk	32wk	1 wk	6 wk	4 mnth	8 mnth	12 mnth
Pregnancy distress	TPDS	X	X	X					
Depression	E(P)DS	X	X	X	X	X	X	X	X
Anxiety	GAD-7	X	X	X	X	X	X	X	X
NVP	PUQE	X							
CTS	BCTQ			X	X	X	X	X	X

BCTQ: Boston Carpal Tunnel Questionnaire; CTS: Carpal Tunnel Syndrome; E(P)DS: Edinburgh (Postnatal) Depression Scale; GAD-7: Generalized Anxiety Disorder scale; NVP: nausea and vomiting during pregnancy; PUQE: Pregnancy-Unique Quantification of Emesis; TPDS: Tilburg Pregnancy Distress Scale.

To measure pregnancy-related emotional distress, the 16-item Tilburg Pregnancy Distress Scale (TPDS) [69] with subscales ‘negative affect’ and ‘partner involvement’ is assessed at each trimester of pregnancy. The TPDS has been validated among Dutch pregnant women and showed good internal consistency overall (Cronbach’s alpha = 0.78), as well as for each subscale (negative affect (TPDS-NA), 11 items, alpha = 0.80; partner involvement (TPDS-PI), 5 items, alpha = 0.80) [69].

Symptoms of depression during pregnancy are measured with the 10-item Edinburgh Depression Scale (EDS), validated in Dutch postpartum women [70, 71] as well as pregnant women [72]. The EDS is a reliable instrument for screening depression in each of the three trimesters of pregnancy. The reliability values of the EDS indicated by Cronbach's alpha-coefficient per trimester are respectively 0.82, 0.83, and 0.84 [72]. The EDS total score ranges from 0 to 30, with higher scores indicating more depressive symptoms. Trimester-specific cut-off points were determined, lower than the commonly applied cut-off in the postpartum period. A cut-off value of 11 in the first trimester and of 10 in the second and third trimesters gives the most adequate combination of sensitivity, specificity, and positive predictive value [72]. The EDS has repeatedly been shown to consist of three subscales: anhedonia, anxiety and depression, with anhedonia and depression assessing different concepts of depression [73]. However, the anhedonia subscale only consists of two items. Therefore, within the HAPPY study, the EDS has been extended with several anhedonia items depicted from the Dutch validated version of the MASQ (Mood and Anxiety Symptoms Questionnaire, originally developed by Watson and Clark in 1991 [74, 75]). Psychometric properties of this extended 14-item EDS-version will be tested in a subsample.

During the postpartum period, the Edinburgh Postnatal Depression Scale (EPDS) is used to assess depressive symptoms at one and six weeks postpartum and at 3, 6 and 12 months postpartum. The 10-item EPDS has been validated among postpartum women in The Netherlands and revealed appropriate psychometric characteristics with a Cronbach’s alpha of 0.87 [70, 71]. The total score ranges from 0 to 30, with higher scores indicating more depressive symptoms. A cut-off value of 9 will be used to detect a possible postpartum depression [76].

The Generalized Anxiety Disorder scale (GAD-7) is a valid and reliable (Cronbach’s alpha = 0.89) device for screening generalized anxiety disorder and for assessing its severity [77, 78]. The GAD-7 consists of seven items with a four-point Likert scale (0 = ‘not at all’ to 3 = ‘nearly every day’). The total score ranges from 0 to 21, with higher scores representing higher levels of anxiety symptoms [77]. Cut-off points of 5, 10, and 15 are associated with respectively mild, moderate, and severe levels of anxiety [78].

To assess the severity of NVP (or ‘morning sickness’) in the early phase of pregnancy, we use the first trimester Pregnancy-Unique Quantification of Emesis (PUQE) scale [2]. The assessment is based on three physical symptoms over the first trimester: nausea, vomiting, and retching. The PUQE, which consists of three quantification items with five answer categories, appears to be a reliable instrument to determine severity of nausea and vomiting during pregnancy [2]. Within the HAPPY study, the Dutch version of the PUQE is assumed to be validated.

To examine CTS, the question: “Did you suffer any of the following symptoms during pregnancy: pain, tingling sensations, or numbness in hands or wrists?” is used including the three key symptoms of CTS. Subsequently, the Boston Carpal Tunnel Questionnaire (BCTQ) is a disease-specific CTS questionnaire that consists of two subscales: the symptom severity scale (11 items) and the functional status scale (8 items). It has been validated in The Netherlands, showing adequate psychometric properties [79]. The BCTQ is assessed at 32 weeks of gestation and retrospectively at one week postpartum to evaluate CTS symptoms during the last eight weeks of pregnancy. Moreover, in order to evaluate the course of symptoms after childbearing it is assessed at 6 weeks, and 4, 8 and 12 months postpartum.

Furthermore, women are asked whether a negative event did occur during the first trimester of pregnancy or during the period between the previous and next assessment until 12 months postpartum.

### Statistical analyses

Statistical analyses will be performed using SPSS 20.0 (Statistical Package for Social Sciences version 20, IBM, Chicago, Illinois, USA). The confirmatory factor analysis will be done using AMOS (version 18, IBM, Chicago, Illinois, USA).

First, we will test for normality and the computation of descriptive statistics. Data will be presented as mean scores ± SD for continuous data and as percentages for categorical data. At a univariate level, differences in mean scores on questionnaires between subgroups (e.g. with and without thyroid dysfunction, preterm birth, or major depression) will be analysed using t-tests (for parametric data) and Mann–Whitney-U tests (for non-parametric data). Significance will be tested two-sided at a significance level of 0.05. Relations between continuous variables will be calculated using Pearson or Spearman correlations.

In order to test the psychometric properties of (partially) new scales (extended EDS, PUQE e.g.), factor analysis will be performed as well as reliability analysis in subsamples. For this purpose, samples of women will be randomly selected by the computer. One sample will be used for explorative factor and reliability analysis (Cronbach’s alpha). A principal component analysis with oblimin rotation and a scree test will be used to select factors for retention. Factor loadings above 0.40 are considered important. The minimum value of the Kaiser-Meyer-Olkin (KMO) index for a good factor analysis is suggested to be ≥ 0.60 [80] and the Bartlett’s test for sphericity value reaches significance at 0.05 level. Internal consistency analyses will be conducted using Cronbach’s alpha for the total scale and possible subscales derived from factor analysis. Cronbach’s alpha reliability statistic of ≥ 0.70 is considered as the minimum acceptable criterion of instrument internal reliability [81]. In a similar second sample confirmative factor analysis (CFA with AMOS, IBM) will be used to test stability of the factor structures as found in the first sample. The comparative fit index (CFI), normed fit index (NFI), and the root mean square error of approximation (RMSEA) are generally considered good parameters to evaluate model fit. Adequate model fit can be assumed with a CFI ≥ 0.80, NFI ≥ 0.80, and RMSEA ≤ 0.05 for good and ≤ 0.08 for adequate fit [82, 83]. Similar procedures will be used for other new questionnaires with the assumption that four to ten participants per item with a minimum of 100 participants are needed for adequate analysis [81, 84].

At a multivariate level, different techniques will be used. In order to evaluate possible causality between different variables path analysis will be performed using structural equation modelling (SEM). For continuous dependent variables (for example PUQE scores or depression scores on the EDS) linear regression models will be used. Stepwise regression analysis will be performed, starting with the independent variable in a first model. Subsequently, block entry with different confounders will be used to calculate the total explained variance (R^2^) and Cohen’s effect size. For these analyses, the EDS scores will be used to define depression as well as the subscales depression and anhedonia. Differences in predicting obstetric outcome will be evaluated for these subscales. With dichotomous variables (preterm birth yes/no e.g.) as dependent variable, single and multiple logistic regression analyses (O.R., 95% CI) will be performed entering the independent variable as well as several confounders into the model.

Regarding CTS, a multiple logistic regression analysis will be performed, with the presence of self-reported CTS (based on the question: “Did you suffer any of the following symptoms during pregnancy: pain, tingling sensations, or numbness in hands or wrists?”) as the dependent variable and fluid retention during pregnancy as independent variable, adjusted for age, parity, BMI, and depression. Furthermore, a multiple linear regression analysis will be performed, with BCTQ score (indicating CTS severity) as dependent variable and fluid retention during pregnancy as independent variable, adjusted for age, parity, BMI, and depression.

Linear mixed effect models will be used to determine the effect of scores on the EDS and the GAD-7 during different trimesters on the request for epidural anaesthesia during labour, obstetric complications, the occurrence of postpartum depression, and the start and continuation of breastfeeding. Linear mixed effect models are able to adjust for missing values and are therefore used to avoid loss of information [85].

For answering the various research questions, different models will be used containing different dependent and independent variables. Confounding variables will be entered into the model according to the literature. The variables age, parity and educational level will be used as confounders in all the models. For example: in examining determinants of start/continuation of breastfeeding during the early postpartum period, obstetric outcome will be entered into the regression as a confounder, while for assessing determinants of CTS, the variable BMI will be included as confounder.

## Discussion

Within the HAPPY study, we aim to evaluate the relationships between several physiological and psychological factors and antenatal and postpartum maternal and infant well-being. The key strength of this large prospective cohort study is the holistic (multifactorial) approach on perinatal well-being combined with a longitudinal design with measurements during all trimesters of pregnancy and the whole first year postpartum. The HAPPY study is unique in several aspects.

First, within one design, a psycho-physiological model to explain NVP will be tested with special attention to HCG and thyroid function. This multifactorial approach to investigate NVP has not yet been reported in literature. Secondly, the prevalence of CTS is assessed using a standardized and validated questionnaire and a possible relation between CTS symptoms and thyroid function during pregnancy will be tested, which has never been reported before. Third, suboptimal maternal thyroid function has repeatedly been associated with poor development of the foetal CNS. However, combining maternal thyroid function with the objective parameters of development of the CNS assessed by ultrasound at 20 weeks gestation has hardly been described. Fourth, within the abundant literature on perinatal depression, there are very few papers looking at the concept of anhedonia and depressed mood, the two key symptoms of depression. Fifth, because The Netherlands have relatively few women asking for epidural anaesthesia during labour (a risk factor for increased instrumental delivery), it is possible to look at psychological determinants predicting a request of analgesia. Finally, the attitude towards initiating and continuation of breastfeeding are assessed, taking into account demographic and psychological characteristics that have repeatedly been assessed during gestation.

Another strength of the study is the large sample size (2,500 participants) which will enable to look at possible relations between psychological and physiological determinants and obstetric complications but which are relatively rare (preterm birth, pre-eclampsia, intrauterine growth restriction). Moreover, the sample size will enable us to validate several questionnaires with explorative and confirmative factor analyses and to use the validated versions in subsamples of HAPPY that are large enough to answer research questions.

The feasibility of HAPPY is guaranteed by the high experience of the perinatal research group of Tilburg University which has an experience of over 20 years of research in this field. During this period several cohorts have been formed showing an overall and stable recruitment rate of over 70% after inviting women through their midwife to participate into pregnancy research [69, 72, 86].

## Abbreviations

BCTQ: Boston carpal tunnel questionnaire
CFI: Comparative fit index
CNS: Central nervous system
CTS: Carpal tunnel syndrome
E(P)DS: Edinburgh (postnatal) depression scale
FT4: Free thyroxine (free T4)
GAD-7: Generalized anxiety disorder scale with 7 items
HAPPY: Holistic approach to pregnancy and first postpartum year
HCG: Human chorionic gonadotropin
HG: Hyperemesis gravidarum
HT: Hypothyroidism
IUGR: Intrauterine growth restriction
NFI: Normed fit index
NVP: Nausea and vomiting during pregnancy
PUQE: Pregnancy-unique quantification of emesis scale
RMSEA: Root mean square error of approximation
SEO: Protocolized ultrasound at 20 weeks of gestation/ structural ultrasound examination
SGA: Small for gestational age
TPDS: Tilburg pregnancy distress scale
TPO-Ab: Thyroid peroxidase antibodies
TSH: Thyroid stimulating hormone, thyrotropin
WHO: World Health Organization.
